# Report of a case of cyberplagiarism - and reflections on detecting and preventing academic misconduct using the Internet

**DOI:** 10.2196/jmir.2.1.e4

**Published:** 2000-03-31

**Authors:** Gunther Eysenbach

**Affiliations:** ^1^University of HeidelbergDepartment Of Clinical Social MedicineUnit for CybermedicineHeidelbergGermany

**Keywords:** Ethics, Professional, Plagiarism, Scientific Misconduct, Publishing, Internet, Retraction of Publication, Copyright, Peer Review, Software, Information Retrieval

## Abstract

**Background:**

The Internet is an invaluable tool for researchers and certainly also a source of inspiration. However, never before has it been so easy to plagiarise the work of others by clipping together (copy & paste) an apparently original paper or review paper from paragraphs on several websites. Moreover, the threshold of stealing ideas, whether lifting paragraphs or perhaps even whole articles from the Internet, seems to be much lower than copying sections from books or articles. In this article, we shall use the term "cyberplagarism" to describe the case where someone, intentionally or inadvertently, is taking information, phrases, or thoughts from the World Wide Web (WWW) and using it in a scholarly article without attributing the origin.

**Objective:**

To illustrate a case of cyberplagiarism and to discuss potential methods using the Internet to detect scientific misconduct. This report was also written to stimulate debate and thought among journal editors about the use of state of the art technology to fight cyberplagiarism.

**Methods:**

A case of a recent incident of cyberplagiarism, which occurred in the *Journal of the Royal College of Surgeons of Edinburgh* (JRCSEd), is reported. A systematic search of the Internet for informatics tools that help to identify plagiarism and duplicate publication was conducted.

**Results:**

This is the first in-depth report of an incident where significant portions of a web article were lifted into a scholarly article without attribution. In detecting and demonstrating this incident, a tool at www.plagiarism.org, has proven to be particularly useful. The plagiarism report generated by this tool stated that more than one third (36%) of the JRCSEd article consisted of phrases that were directly copied from multiple websites, without giving attribution to this fact.

**Conclusions:**

Cyberplagiarism may be a widespread and increasing problem. Plagiarism could be easily detected by journal editors and peer-reviewers if informatics tools would be applied. There is a striking gap between what is technically possible and what is in widespread use. As a consequence of the case described in this report, JMIR has taken the lead in applying information technology to prevent and fight plagiarism by routinely checking new submissions for evidence of cyberplagiarism.

## The JRCSEd Cyberplagiarism Case: Chronology of Events

**Figure 1:**
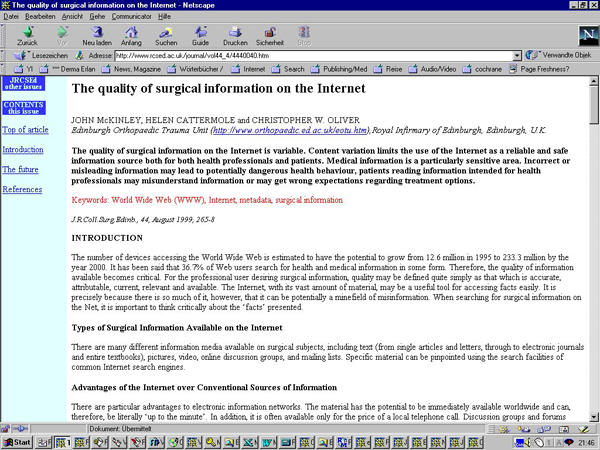
The online version of the questionable article, which contained lifted phrases from the web, as published in the Journal of the Royal College of Surgeons of Edinburgh

On 5 August 1999, a paper titled "The quality of surgical information on the Internet" (see Figure 1) was published in the *Journal of the Royal College of Surgeons of Edinburgh* (JRCSEd) [1]. The JRCSEd is a journal indexed in several bibliographical databases including Biological Abstracts, EMBASE, Current Contents, Index Medicus/MEDLINE, and others; and is published bi-monthly by the Royal College of Surgeons of Edinburgh.

After publication, it was determined that more than one third (36%) of this article consisted of phrases that were directly copied from multiple websites, without giving attribution to this fact. This can be labelled as plagiarism, which has been defined by the US Committee on Science, Engineering, and Public Policy as "using the ideas or words of another person without giving appropriate credit." The Committee continues by saying that plagiarism is a "strike at the heart of the values on which science is based. These acts of scientific misconduct not only undermine progress but the entire set of values on which the scientific enterprise rests" [2]. In this article, I will refer to this incident as an act of cyberplagiarism alluding to the fact that information from "cyberspace" (the Internet) was lifted into a scholarly article. The question of whether this incident of plagiarism was intentional or inadvertent should not be discussed here.

### The case of cyberplagiarism

The following is a quick recap of the event: Shortly after publication of the article in question [1], the senior author (C.O.) announced the publication of the paper by sending out emails alerting those potentially interested in this paper; among the recipients was the author of this report (G.E.), who immediately recognised that significant parts of this paper were based on ideas presented in the article "Towards quality management of medical information on the internet: evaluation, labelling, and filtering of information," published a few months earlier in the *British Medical Journal*(BMJ) [3]. This paper was not cited. This fact alone would probably not have fostered any further action taken alone, as probably every published researcher has experienced a similar situation.

**Figure 2:**
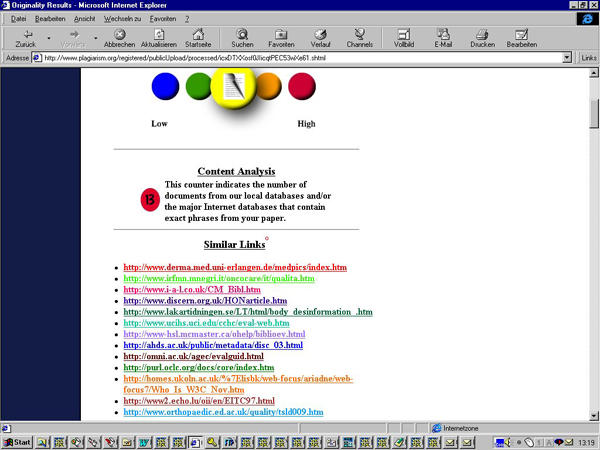
The plagiarism.org report detected similarities with twelve webpages (listed under "similar links"). The originality of the paper was rated as "medium."

But in this case it went beyond just a missed reference, as the authors of the JRCSEd article also took material from the website http://medpics.org(which was then posted on the site http://www.derma.med.uni-erlangen.de/medpics/) without attribution. More than half of the abstract consisted of sentences from this website, and also a subjective expression of an opinion ("We believe that one of the responsibilities of any health professional is to guide patients through health related information") was lifted unchanged from the website into the article. In total, at least three lengthy paragraphs (about 400 words, which constituted around 20% of the article) were taken practically unchanged from the medPICS website. A later comparison with the whole Internet using the tool at http://www.plagiarism.org(described below) identified further portions of the manuscript which had been taken from other websites, most notably another 350 words lifted without attribution from http://purl.oclc.org/docs/core/index.htm, a webpage copyrighted by the Dublin Core Metadata Initiative. Taken together, well over one third (36%) of the manuscript published in JRCSEd consisted of sentences that had been lifted directly and without editing from other websites, (see plagiarism.org report, Figure 2) without attribution or giving credit to the originators.

The editor in chief of JRCSEd, Professor Oleg Eremin, alerted by the author of this report (G.E.), started an investigation. The editorial board concluded that "there has been a serious infringement of copyright." The electronic version of the article was permanently deleted from the journal website. The author of the plagiarism (C.O.) was asked to write a letter of apology for publication. In a subsequent issue of JRCSEd, the editorial board published a notice of the fact that parts of the manuscript were identical to online material published at http://medpics.org and about the withdrawal of the article [4]. C.O. himself apologized in a published letter [5] acknowledging that "my article contained certain paragraphs which are broadly similar to articles which he has published at http://medpics.org and which I did not reference in my paper" and saying that "it was entirely unintentional and occurred as a result of an oversight in the preparation of the manuscript." No reference was made to the fact that more than one third of the article was actually not only "broadly similar" but indeed taken unchanged from the web, nor to the fact that not only one website was exploited that way.

## Using Informatics Tools and the Internet to Detect Plagiarism

The Internet, with its vast amount of information at the fingertips of every researcher, makes it easy to lift whole phrases and paragraphs into scholarly articles. This can be a useful strategy to gather material and ideas; such techniques, and also quotes from websites, are certainly legitimate, as long as the sources are acknowledged and quotes are clearly identified as such. As this case shows, researchers are not always successful in quoting properly and may even inadvertently end up committing plagiarism.

Luckily, the Internet can also provide some technical solutions for researchers to identify unintentional omissions of attributions and for journal editors and peer-reviewers to detect and fight plagiarism. Although a number of informatics approaches are thinkable and could be applied routinely, not all of the possible approaches are in fact realised in the form of commercially available applications, and if they are, they are rarely used by researchers, journal editors, or peer-reviewers. In the following sections, I will review a number of possible approaches (which in part still wait for programmers to translate them into software).

### Retrospective control: Checking submitted manuscripts against the web or a collection of articles

One possible approach is to check a manuscript (for example a manuscript that has been submitted to a peer-reviewed journal) against the whole World Wide Web (WWW) and/or another collection of published articles (such as the abstracts in MEDLINE, the full text articles in PubMed Central, or e-print servers), in order to identify similar or identical phrases. While generic search engines such as AltaVista could be used to search for simple phrases, they do not allow the user to check a whole manuscript against the Web. Moreover, they cannot detect simple word substitutions; thus, plagiarists may hide the true origin of their selections by simply replacing as many words as possible with synonyms.

A more sophisticated, specialized "search engine" to detect plagiarism has been developed by Barrie and Presti [6]: http://www.plagiarism.orgwas originally developed for professors to check the originality of student term papers. Term papers submitted for a class requirement can be checked against a database of other manuscripts collected from different universities, classes, and from all over the Internet.

To test the power of the system I submitted the questionable manuscript published in JRCSEd (see case report above) to the system. The plagiarism report was returned within 24 hours. The system not only flagged the paper as "medium original," but also highlighted 36% of the document as originating from different websites, most notably from the med-PICS and the Dublin Core metadata websites (see Figure 2,Figure 3, and Figure 4).

**Figure 3a:**
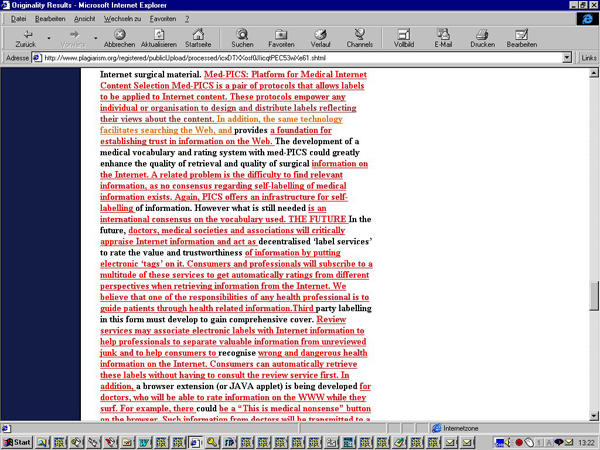
Fig. 3a+b. The words which are underlined and highlighted red in the plagiarism.org report (a) were lifted from the website medpics.org (b)

**Figure 3b:**
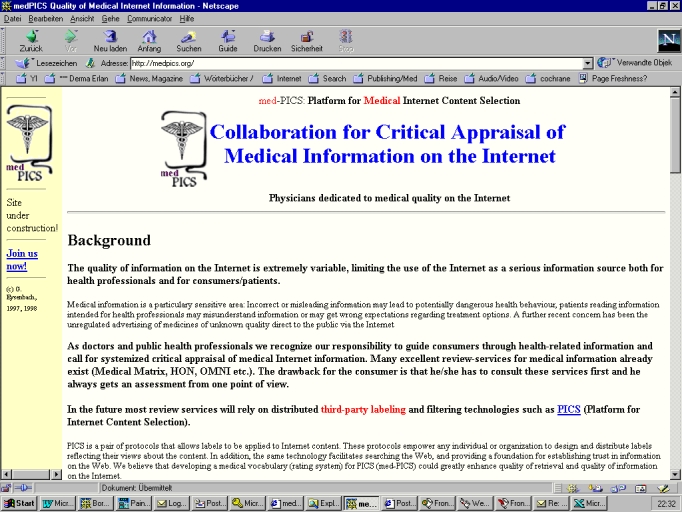
Fig. 3a+b. The words which are underlined and highlighted red in the plagiarism.org report (a) were lifted from the website medpics.org (b)

**Figure 4a:**
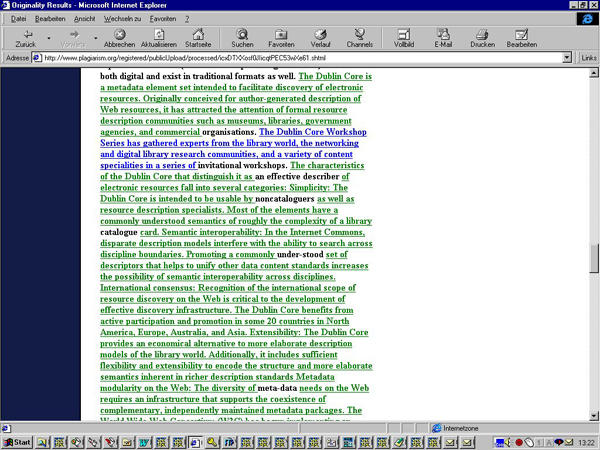
Fig. 4 a+b. The words which are underlined and highlighted green in the plagiarism.org report (a) were lifted from the Dublin Core metadata website (b)

**Figure 4b:**
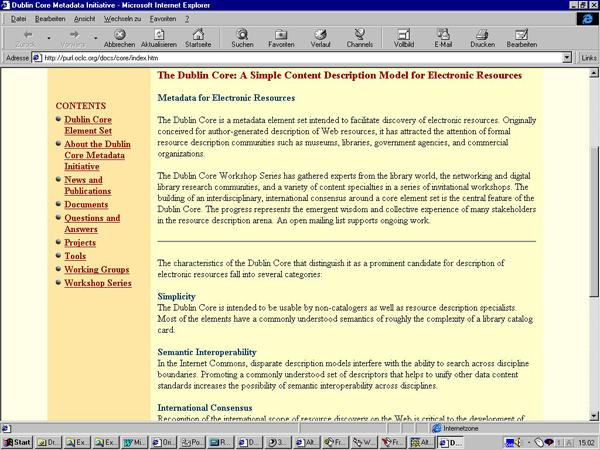
Fig. 4 a+b. The words which are underlined and highlighted green in the plagiarism.org report (a) were lifted from the Dublin Core metadata website (b)

As an aside it should be noted that the plagiarism.org tool proved to be very sensitive, in that it also retrieved several websites which cited the same or a similar set of publications. Thus, a tool like plagiarism.org could also be used to identify similar publications on the Web which deal with related topics; thus it may serve a similar function as the "Related Articles" button in PubMed [7].

### Prospective monitoring

Other scenarios could be imagined, but are not yet available. For example, one possible future development could be that Web authors would be able to use special search engines to monitor the Web (or full text databases) prospectively and continuously to receive alerts when there have been parts of their documents "webnapped," i.e. published on other websites or lifted into articles. This would require that the authors submit whole published manuscripts or register the URL at a special search engine, together with their email address. The search engine would then not only crawl and index webpages like a normal search engine, but also automatically notify Web authors if a "similar" page shows up somewhere on the Web, or if a similar article appears in a dynamic database such as PubMed Central, Medline, in e-print servers, or other databases containing full text articles or abstracts. In fact, such software agents would not only be useful in detecting plagiarism, but could also be used to alert authors of similar new articles in their field being published on the Web or in the literature.

### Detecting duplicate publication

Plagiarism comes in many different varieties. When authors "plagiarize" themselves this is called "redundant" or "duplicate publication." According to Charles Babbage, from his book *Reflections on the Decline of Science in England*(cited in Lock [8], p 161), this belongs to the category of "Trimmin and Cooking" (while plagiarism is classified as "fraud"). The Uniform Requirements For Manuscripts Submitted To Biomedical Journals of the International Committee of Medical Journal Editors (ICMJE) [9] state that:

Readers of primary source periodicals deserve to be able to trust that what they are reading is original, unless there is a clear statement that the article is being republished by the choice of the author and editor. The bases of this position are international copyright laws, ethical conduct, and cost-effective use of resources.

Duplicate publication is another kind of misconduct which could be detected by the use of modern information technology: Stephen Lock already noted that "duplicate publication might be disclosed more often if journal offices were to routinely search the databases" [8, p 162]. Never before has it been easier to compare each submitted article against the Internet and databases such as PubMed Central and MEDLINE to detect cases of duplicate publication.

#### An example: Redundant publication of a "letter to the editor"

Interestingly, a case of duplicate publication occurred in the very same issue of the very same journal, conducted by the very same person as described in the case above: On page 278 of JRCSEd, C.O. published a letter "How to cope with unsolicited Email from the general public seeking medical advice" [10], which was nearly identical to a letter submitted to BMJ by the same author, entitled "Automatic replies can be sent to unsolicited email from general public," published on 27 November 1999 [11]. The titles of the letters are different, as they are usually changed by the editorial staff - the original letter submitted to BMJ as a rapid reply did have the same title as the letter submitted to JRCSEd. (As an aside it should be mentioned that, interestingly, the authors are not exactly the same - the JRCSEd piece lists one more author. A case of gift authorship?) As in neither of the journals did a note appear pointing to the fact that the letter also appeared in another journal, this meets the definition of duplicate publication, which is, according to the international guidelines of ICMJE cited above, considered unethical. According to an informal survey among editors of the World Association of Medical Editors (WAME), there is consensus among editors that the same rules apply for letters as apply for articles: Duplicate publication should be disclosed to editors.

Without discussing this case further at this point, it should only be mentioned that intelligent software agents could be developed to alert journal editors about possible cases of redundant publication and copyright violations by automatically comparing publications with each other - for example within and between PubMed Central, Medline, in e-print servers and the web - and alert publishers if similarities are found. As both JRCSEd and BMJ have online versions of their journals, an intelligent software agent could have detected this case of duplicate publication. Once again, the effect of installing and applying such systems would be primarily an educational one: If such measures were in existence and their use known, this would probably discourage authors from submitting redundant articles and committing plagiarism.

### Software that analyzes writing styles

It should be noted that other informatics techniques for detecting plagiarism exist. The Glatt Plagiarism Screening Program is a computer program especially targeted for teachers who want to prove the guilt or innocence of a student. The program detects plagiarism by analysing the writing style within a document. The software developers say that each person has an individual style of writing which is as unique as fingerprints. The procedure is described as follows: "The Glatt Plagiarism Screening Program eliminates every fifth word of the suspected student's paper and replaces the words with a standard size blank. The student is asked to supply the missing words" [12]. Thus, basically this is a test of memory for the student's own writing style - it is assumed that authors know and can remember their own writing better than anyone else. The number of correct responses, the amount of time intervening, and various other factors are considered in assessing the final Plagiarism Probability Score. The authors claim that the program has a specificity of 100% ("no student has been wrongly accused"). The description of this approach makes clear that this program is less suitable for screening and comparing large amounts of documents, but more appropriate to proving plagiarism in an individual case.

### Tools to detect software plagiarism

As an aside, it should also be briefly mentioned that in the field of software development and informatics education, several tools are available which can test the similarity of software to protect computer codes from being lifted; examples include the software similarity tester SIM [13] and software named "MOSS" (Measure Of Software Similarity), which looks for similar or identical lines of code sprinkled throughout a program, then creates a web page where the instructor can see the top 40 matches [14].

### Metainformation and hidden watermarks

The future may bring even more possibilities, especially helping authors avoid inadvertent plagiarism. One option would be to expand the concept of "copy & paste" towards "copy & paste & attribute (=give credit to the source)." Future versions of word processors could be designed which allow authors to clearly identify which parts of the document have been inserted by copy & paste and where they come from. For example, authors could be able to click on the text and the word processor would show in a comment field from what website (or other application) this "copied & pasted" part originated from.

Other developments may include techniques to assign invisible metainformation to electronic information, which could identify the author and which cannot be stripped. Such invisible "watermaters" are already in use for digital images, but future operating systems may also support metainformation assigned to text, so that the author of a given paragraph could be identified, even if the text is "copied and pasted" from one application into another.

On a different level, the company Xerox is also active in developing products which make redistribution of digital content impossible. The Digital Property Rights Language (DPRL) is a computer-interpretable language, developed at the Xerox Palo Alto Research Center, which "describes distinct categories of uses for digital works in terms of rights, including rights to copy a digital work, or to print it out, or to loan it, or to use portions of it in derivative works" [15]. DPRL is not a document protection technology. Protection of content integrity and the persistent control of digital property rights is accomplished through the use of The Xerox Self Protecting Document (SPD) [16]. "SPD contains the encrypted content, rights associated with it, watermarks, usage policies and a set of controls that travel along with the document in the form of Java applets. Proven cryptographic algorithms ensure complete protection during rendering by converting a document to the rendered form in various stages; thus, intercepting the document at any stage will not yield a usable form of the document."

### Another form of academic misconduct: Underreporting of research

Not only plagiarism and duplicate publication ("overreporting of research") can be a problem in medical science; "," i.e. not publishing the results of a randomised controlled trial, has also been called scientific misconduct [17]. The reason for this is that the biggest threat of a systematic review and meta-analysis is publication bias. Reviewers and policy makers need a complete picture of the results of all randomised controlled trials conducted, and not only of positive or interesting trials, which have been published by researchers. We have recently shown that the Internet is useful in identifying unpublished and ongoing trials, and suggested specialised search engines and software agents that collect information about ongoing trials on the Internet [18]. In addition to prospective trial registers [19], such a search engine could help to detect the digital traces most researchers leave today on the Internet when they conduct a study, such as hints to grant proposals or webpages for recruitment of participants. This would aid reviewers in locating unpublished studies and at the same time - if sanctions for this kind of scientific misconduct are in place - discourage researchers from leaving clinical studies unpublished.

## Reasons for and Prevalence of Plagiarism

Many authors seem to be encouraged to copy from the web as electronic publications are seen as "inferior" in quality and worthiness of protection, and are seen as more volatile than "real" publications on paper. While the majority of authors would refrain from copying whole paragraphs from printed articles, the barrier to do the same from web publications seems to be lower, as information on the web would disappear sooner or later, making the proof of plagiarism apparently impossible, while the printed journal would remain in the library as a durable witness of plagiarism waiting to be discovered and used as evidence. However, plagiarists should be warned that material on the Internet is not as volatile as they may think, and that future historians will well be able to reconstruct online-plagiarism, as there are online-archives of the Internet such as http//:www.archive.org [20].

Insufficient familiarity with English [21], the pressure to publish much and fast [8], and sometimes also sloppiness and forgetfulness are probably the main reasons for cyberplagiarism. An interesting question is how common plagiarism, especially "cyberplagiarism," actually is. Interestingly, questioned about the case described above, C.O. was quoted in Nature as saying: "If you ran [this system] on every article [in the medical literature] that comes out, you would find this happening all over the place" [22].

Jeremy Wyatt, a respected medical informatics researcher from London and an editorial board member of the Journal of Medical Internet Research, also says that he has "seen paragraphs of my work copied in other people's papers without acknowledgement at least three times now (in obscure conference papers and medical informatics journals) but have never kept a note of it; after the initial anger, I dismissed it as a case of "imitation is the sincerest form of flattery'." Future studies applying tools such as plagiarism.org in editorial offices may establish estimates on how widespread this phenomenon is.

### JMIR the first scholarly journal to screen submitted manuscripts for plagiarism

In the future, the Journal of Medical Internet Research will routinely check accepted manuscripts for plagiarism, using the automatic plagiarism detector at plagiarism.org. We are the first scholarly journal worldwide to adopt such a plagiarism screening policy, but we hope (and expect) that other biomedical journals will follow. Authors should remember that there is only one easy and reliable way to avoid plagiarism charges: that is to cite the source properly, even if it is "only" an electronic document [23].
